# Monitoring of Environmental Hg Occurrence in Tunisian Coastal Areas

**DOI:** 10.3390/ijerph18105202

**Published:** 2021-05-13

**Authors:** Amel Jebara, Vincenzo Lo Turco, Caterina Faggio, Patrizia Licata, Vincenzo Nava, Angela Giorgia Potortì, Rosalia Crupi, Hedi Ben Mansour, Giuseppa Di Bella

**Affiliations:** 1 Research Unit of Analysis and Process Applied to Environmental, APAE UR17ES32 Higher Institute of Applied Sciences and Technology Mahdia, University of Monastir, Monastir 5000, Tunisia; ameljebaraa@gmail.com (A.J.); hdbenmansour@gmail.com (H.B.M.); 2 BioMorf Department, University of Messina, Polo SS Annunziata, 98168 Messina, Italy; vloturco@unime.it (V.L.T.); vnava@unime.it (V.N.); agpotorti@unime.it (A.G.P.); gdibella@unime.it (G.D.B.); 3 Department of Chemical, Biological, Pharmaceutical and Environmental Sciences, University of Messina, 98122 Messina, Italy; cfaggio@unime.it; 4 Department of Veterinary Science, University of Messina, Polo SS Annunziata, 98168 Messina, Italy

**Keywords:** Mahdia coastal, Hg, *Posidonia oceanica*, *Sarpa salpa*, *Sparus aurata*, tolerable weekly intake

## Abstract

Total mercury (Hg) was determined in 450 environmental samples (seawater, sediment plant and fish) from five Mahdia coastal areas (Tunisia). Tolerable Weekly Intake% (TWI) values, according to the European Food Safety Authority (EFSA), were calculated based on the average metal concentration in fish and the average weekly fish consumption rate. Hg was accumulated mainly in fish and in *Posidonia oceanica* leaves. Hg in sediment ranged from 1.88 μg/kg dry weight (d.w.) to 7.48 μg/kg d.w., while it was between 0.32 μg/kg and 0.19 μg/kg in seawaters. Our study showed high concentration in *Posidonia oceanica* in S3 (plant = 16.76 ± 4.48 μg/kg d.w.) as compared to those in S4 sites (plant = 5.33 ± 0.05 μg/kg d.w.). Concentrations for *S. aurata* and *S. salpa* in the Rejiche area exceeded the EC 1881/2006 legislation with values of 1.9 mg/kg and 2.5 mg/kg, respectively, and consumers may be exposed to high concentrations of Hg that exceeds the EFSA. The results showed that the fish species should be constantly monitored due to their TWI% of 154.5% for *S. aurata* and 209.8% *S. salpa* respectively.

## 1. Introduction

Mercury is one of less abundant elements in the Earth’s crust and it is contained in the biosphere due to human activities. It is a common environmental toxin in the general environment with growing importance in recent years [1,2,3]. This element, through soil and contaminated water, enters the food chain by means of lower microorganisms, with poorly characterized and varied mechanisms between ecosystems, thereby polluting plants and animals. Aquatic organisms are the main source of poisoning in humans by Hg and the mechanisms underlying this process are not yet known; some fish, in particular, tend to have the highest bioaccumulation rates of this element [4,5,6,7,8]. It has been established that it is not only harmful to the health of vulnerable populations such as pregnant women and children, but is also toxic to normal adults in various ways. Recent studies consistently suggest that chronic exposure, even to low Hg levels, can cause cardiovascular toxicity, reproductive and developmental toxicity, neurotoxicity, nephrotoxicity, immunotoxicity, and carcinogenicity [9]. Once absorbed in our body, it is preferably bioaccumulated in the kidneys, liver and especially in the brain, in the latter estimated to have a half-life of almost 20 years, and the major source of Hg intake is food [10]. Toxic effects in species of mammals have been observed in association with local sources of Hg release in the environment [11].

Toxicological tests have demonstrated the toxic effects of chronic exposure to Hg in the reproductive system, immune system, nervous system, cardiovascular system, as well as to the fetus and demonstrated cellular toxic effects in diseases such as leukemia and Hodgking’s. When inorganic Hg reaches the aquatic environment, it is methylated by both biotic and abiotic methylation to produce methylmercury (CH_3_Hg^+^), which can be bioaccumulated and biomagnified into the biota [3,12,13,14,15].

The most important source of Hg food poisoning in humans is methyl Hg and dimethylmercury present in fish tissues and it has the ability to bind with the -SH groups of proteins as well as microsomal and mitochondrial enzymes. This mechanism of action causes a form of both acute and chronic toxicity, nonspecific damage and cell death.

Organic Hg is highly lipophilic and crosses the gastrointestinal mucosa very easily with a bioavailability of 90–95%. In case of acute intoxication, the effects observed are bronchopneumonia, with neurological symptoms in the central nervous system and gastrointestinal tract. In chronic toxicity, the effects found include tremors, hallucinations, kidney damage, neurotoxicity in the cerebral cortex (one of the areas of the brain responsible for cognitive activity) and the cerebellum (part used in motor activity), changes in movements, muscle weakness, loss of vision and hearing, and death [13,14,15].

Fish accumulate Hg through the consumption of water and aquatic organisms and slow elimination of Hg, combined with continuous exposure, causes a significant accumulation of Hg along the food chain [5]. Dietary fish consumption is the main route of human exposure to Hg, a powerful neurotoxin that can significantly compromise human health [16,17,18,19]. The health risks related to the consumption of fish contaminated with Hg are subject to the regulation introduced by many countries and government agencies. Commission regulation of the European Union (EC, 1881/2006) established maximum levels (ML) for the total content of mercury (Hg) in fish, for *Sparus aurata* and *Sarpa salpa* the limit is 0.5 mg/kg wet weight (w.w.) [20,21].

To date, few studies have focused on Hg contamination in the aquatic biota of the southern coast of Tunisia [22,23]. This work is the first study entirely focused on the evaluation of Hg concentration in sediments, seawater, *Posidonia oceanica* leaves, *Sparus aurata* and *Sarpa salpa* fish species from the different areas of Mahdia coast (Tunisia). Total Hg concentrations were evaluated in the edible tissues of fish important in the diets of humans in the area of study. Mercury contents were also used to calculate the estimated weekly intake for each species and compared with the Tolerable Weekly Intake (TWI) established by EFSA guidelines (EFSA 2012) to underline the potential health risk for consumers.

## 2. Materials and Methods

### 2.1. Sampling Area

**Figure 1:**
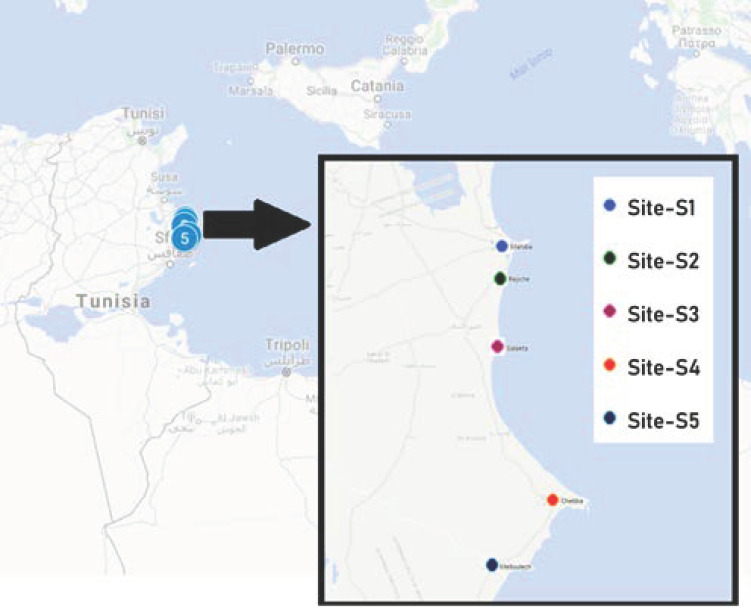
Sampling sites.

The area under investigation was characterized by the presence of a number of factories operating in various highly polluting sectors. In the air, the Hg concentration could be high due to the emission of Hg by industries that use metal in their production processes and this could have contaminated agricultural land, and surface and underground waters [24]. Even in different areas of Tunisia, it was shown that environmental pollution contributes to the release of Hg, thus increasing the contamination level [23,25,26]. The anthropic contamination has direct effects on sediments and waters that affect the resident biota associated and has potential effects on human health. Thus, in order to monitor the environmental Hg occurrence in Tunisian coastal areas, the experimental sampling strategy included five groups of samples: (seawater, sediments, *Posidonia oceanica* leaves, *Sparus aurata* and *Salpa sarpa* muscle tissues). The five areas involved in the study are: S1 Mahdia (35°29′41.43″ N; 11°04′0.64″ E) near aquaculture industry, fishing harbour and in front of a nautical centre working just in Summer; S2 Rejiche (35°28′36.321″ N; 11°03′12.882″ E) is closed by industrial discharges of Mahdia-Rejiche; S3 Salakta (35°19′452.723″ N; 11°02′24.423″ E); S4 Chebba (35°13′44.077″ N; 11°09′40.315″ E) separated from the port to 200 m by a stream of water; S5 Melloulech (35°08′54.916″ N; 11°02′59.98″ E) near a shrimp farming industry. All five sites are located in the Mahdia coastal areas, east Tunisia (Figure 1). A total of 450 environmental samples (165 seawater, 165 sediments, 60 *Posidonia oceanica* leaves, 30 *Sparus aurata* muscle tissues and 30 *Salpa sarpa* muscle tissues) were collected from five sites (S1, S2, S3, S4, S5) during year 2018–2019.

### 2.2. Sample Collection

The two fish species (*Sparus aurata* and *Salpa sarpa)* were collected from professional fishermen by using a thrown fishing net. The fish species caught were commercially important species. The average length of *Sparus aurata* and *Salpa sarpa* was 25.5 and 21.4 cm, whereas the average weight was 155.8 and 212.6 g, respectively. After taking the measurements, fish samples were washed with deionized water, sealed in poly-ethylene bags and kept in a freezer at −20 °C. After thawing, a portion of the muscle tissue was removed, identified, placed in sterile tubes, and stored in liquid nitrogen. From these, muscle samples were ground in mixer and each fish sample was freeze-dried and then frozen at −80 °C.

The superficial sediment samples were collected with the Veen Grab Sampler (126 cm^2^ area) operated by hand, placed in sterilized plastic bags and stored at 4 °C until analysis. After this step, 10 g sediment samples (w.w.) were oven dried at 35 °C for 48 h [27].

Seawater was collected using acid-washed Niskin-Type general purpose, plastic water samples (2.5 L model; Hydro-Bios Inc., Altenholz, Germany) at depths of 20 ± 3 m. Teflon bottles and silicon tubing were washed using 37% ultra-pure, trace metal grade HNO_3_ and were rinsed three times using Milli-Q deionized water. Teflon bottles and Niskin type bottles were dried in an EPA clean 100 room under a laminar flow hood. Seawater was then acidified using 0.5% ultrapure HCl and placed in a refrigerator at 3 °C until analyses.

*Posidonia oceanica* was collected at 20 ± 3 m depth by scuba divers. At each station, three plants were sampled. After collection, the plants were immediately transported, in polyethylene bags, to the laboratory. They were divided into the following fractions: roots, rhizomes and leaves. In addition, the leaves were divided into green leaf (upper 3 cm) and basal part (bottom 3 cm). Only mature leaves of comparable length were selected for seagrass analysis. For each station, all roots, rhizomes, green leaf and basal part of the leaves were pooled into four samples. Each sample was lyophilized and then ground in a mortar and pestle to a coarse powder.

Samples of fish, plant, sediment and seawater were analysed in triplicate.

### 2.3. Hg Analysis

Samples (100 mg) were analyzed for Hg concentration using an DMA-80 (Milestone, Shelton, CT, USA) by thermal decomposition (from 60 to 650 °C for about 5–6 min), in oxygen or air atmosphere according to US EPA 7473 method [28].

Prior to the analysis, the calibration curve was constructed using Hg 1000 mg/L certified standard (CZECH Metrology Institute Analytika). The calibration standard solutions were prepared in glass flasks at 5 concentration points, in the range between 0.05 and 10 mg/L. Each standard solution was injected six times. The method was validated according to the EURACHEM guidelines (EURACHEM 2014). The evaluation of the linearity was based on the determination of the coefficient of determination (r^2^). Good linearity was observed, achieving a r^2^ = 0.9996. The detection limit (LOD) and quantification limit (LOQ) were counted as 3 times and 6 times the standard deviation for blanks and were 0.29 µg/kg and 0.58 µg/kg, respectively. For seawater matrix LOD and LOQ were 0.05 and 0.16 µg/L respectively. The accuracy of the Hg analysis was tested using spearfish sample (TB 149) and coastal seawater (IRMM BCR-579) certified reference material (CRM). Recoveries were 93% and 95%, respectively.

## 3. Results

**Figure 2:**
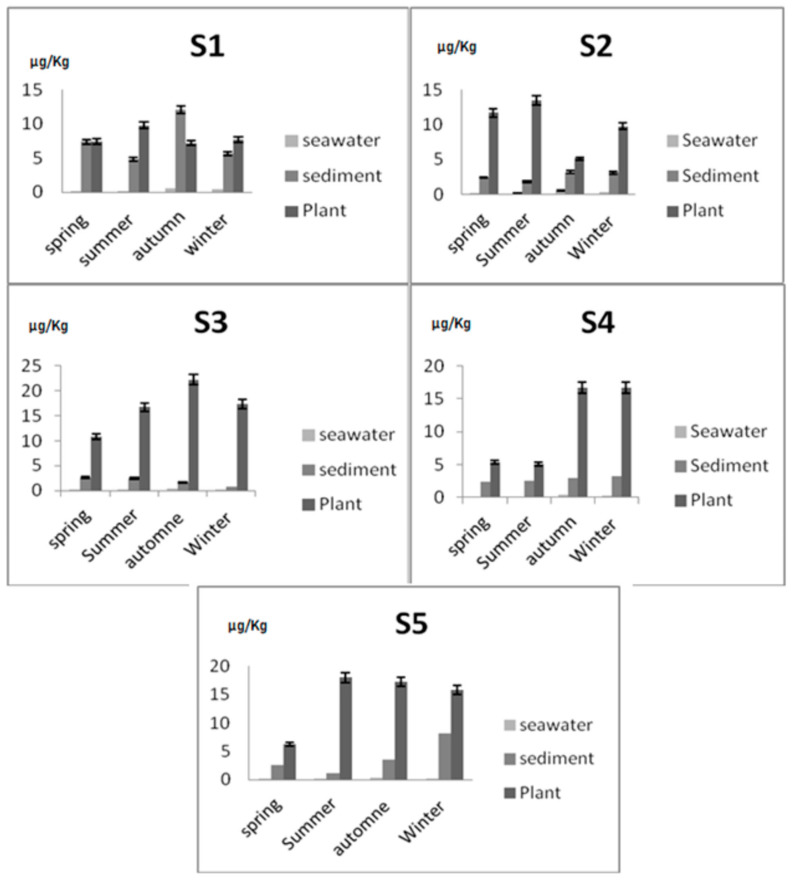
Hg concentration in some environmental samples (seawater, sediment, plant) from different sites according season (μg/kg).

Seasonal variations of Hg concentrations in environmental samples were shown in Figure 2. In the majority of cases, the variation among seasons was different between samples and appeared to depend on the sampling site. Thus, the metal concentration decreased in the following order: *Posidonia oceanica* > sediment > seawater. With the only exception in autumn at the Mahdia site where Hg content was higher in sediment than in plant species (Sediment = 12.1 μg/kg d.w. > Plant = 7.2 μg/kg d.w.). The sediments showed an abundant fine clay component which has a known absorbing capacity due to the content of organic matter. According to the literature, in some stations the trace element concentrations range seasonally in seagrass tissues, with lower values seen during the growing season (September to November) than during the dormancy period. In this area, the main contributors of Hg pollution could be populated cities, domestic discharged and also industrial and agricultural waste waters. In the S2, we observed an apparent decrease in the concentration of Hg in seawater, sediments and plants in autumn (seawater = 0.50 μg/kg; sediment = 3.19 μg/kg d.w.; plant = 5.09 μg/kg d.w.), as well as in the S5 in spring (seawater = 0.40 μg/kg); sediment = 2.52 μg/kg d.w.; plant = 6.27 μg/ kg d.w.). In S3, the results showed no seasonal variation, while S4 demonstrated low Hg content during the spring and summer seasons. The comparison of samples showed that Hg concentration was different among the sampling locations. The concentration of Hg was preferentially accumulated in *Sparus aurata*, *Sarpa salpa* and *Posidonia oceanica* leaves and decreased in the following order: fish > plant > sediment > seawater (*S. aurata* = 878.2 ± 447.6 μg/kg d.w.; *S. salpa* = 607.5 ± 147.9 μg/kg d.w.; Plant = 11.5 ± 2.8 μg/kg d.w.; Sediment = 4.30 ± 3.02 μg/kg d.w.; seawater = 0.34 ± 0.14 μg/kg) (Table 1). In general, the highest Hg content in fish were detected in S2 (*S. aurata* = 1853.7 ± 655.6 μg/kg d.w.; *S. salpa* = 2518.7 ± 231.8 μg/kg d.w.) (Table 1); this value may be due to the wastewater from the sewage drains that polluted the seawater up to 200 m from the beach (WMC/TAP 2019). Few studies found that trace element concentrations in seagrasses were not significantly correlated with column waters [29]. Although seagrasses were able to uptake trace elements from water as well, our study showed high concentration in *Posidonia oceanica* was detected in S3 (plant =16.76 ± 4.48 μg/kg d.w.) than those in S4 (plant = 5.33 ± 0.05 μg/kg d.w.) and it was due to the localization of S3. It was observed that the site studied was likely to be a place of clandestine dumping of waste water from domestic use, they may be providers of septic tank cleaning services. Nevertheless, the possibility that this contamination was due to the abundant presence of fish farming cages in the surrounding maritime areas must be considered and it should also be noted that the presence of toxic elements was less likely to be inherent to pollution from the Salakta port (Figure 2). Concentrations of Hg in sediment ranged from 1.88 μg/kg d.w. at site 3 to 7.48 μg/kg d.w. at site 1, on the other hand, Hg concentrations in seawaters was between 0.32 μg/kg at site 1 and 0.19 μg/kg at site 5 (Table 1). The high Hg concentration found in the seawaters samples from site 1 could be affected by waste from port activity which includes repair shops such as naval engineering, electronics and electricity, as well as carpentry. The Hg concentrations (μg/kg) in *Sparus aurata* and *Sarpa salpa* muscle tissues were showed in Table 1. Overall, Hg values ranged from 438.5 to 1853.7 μg/kg for *Sparus aurata* and 14.1 to 2518.7 μg/kg for *Sarpa salpa* species. The variability of Hg levels among the different sites studied is in accordance with the process of the uptake of this metal in fish and the interaction of numerous parameters, either abiotic (water and sediments) or biotic (size, sex, longevity, growth rate, feeding habits, trophic position, habitat). The results of this investigation indicated, however, markedly higher values of Hg in fish species from site 2, suggesting that the variability observed between different environmental samples may reflect variability of Hg levels found in the environment.

The Tolerable Weekly Intake (TWI)% values, according to the EFSA, was calculated based on the average concentration of metal in fish tissue (after converting the values from dry weight to wet weight) and the average weekly fish consumption rate. The risk exposure to total Hg was determined for an average serving portion of 200 g of fish in a week for a 60 kg adult, as TWI% of 4 mg/kg b.w. recommended by EFSA.

The ranges of Hg contents and their relative TWI% value calculated in this study were reported in Table 1. The weekly consumption of 200 g of the fish species by a 60 kg of body weight point out a health risk of exposure to the toxic effect of total Hg for *Sparus aurata* and *Sarpa salpa* from site 2, as the TWI% value was exceeded in these fish samples in the areas of the Mahdia coastal, no risk was observed for samples from other sites.

## 4. Discussion

### 4.1. Hg Correlation between Samples and Sites

**Figure 3:**
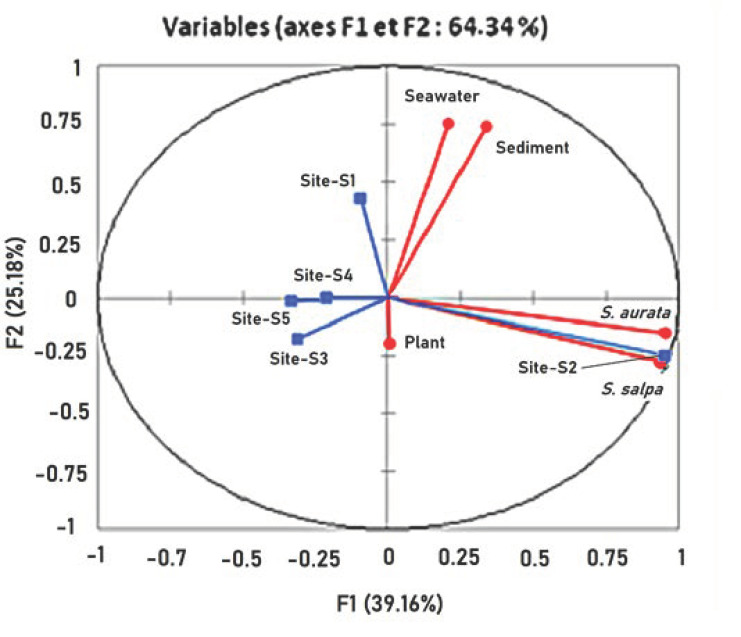
Correlation circle between samples (seawater, sediment, plant) and sites.

Principal Component Analysis (PCA) was used to elaborate some possible correlations between Hg concentration on seawater/sediment and fish/plant. In regard to the collection sites, the Hg concentration ranged in different environmental samples from five stations of Mahdia littoral and it could be represented with two main axes. The two main components account for 64.34% of the total variance. The first factorial plane that accounts for 39.16% of the variance was characterized by high positive correlation between *Sparus aurata* and *Sarpa salpa*. At the same time, 25.18% of the second factorial plane of the variance represented high positive loads of seawater and sediment but a negative contribution of plant (Figure 2 and Figure 3).

**Figure 4:**
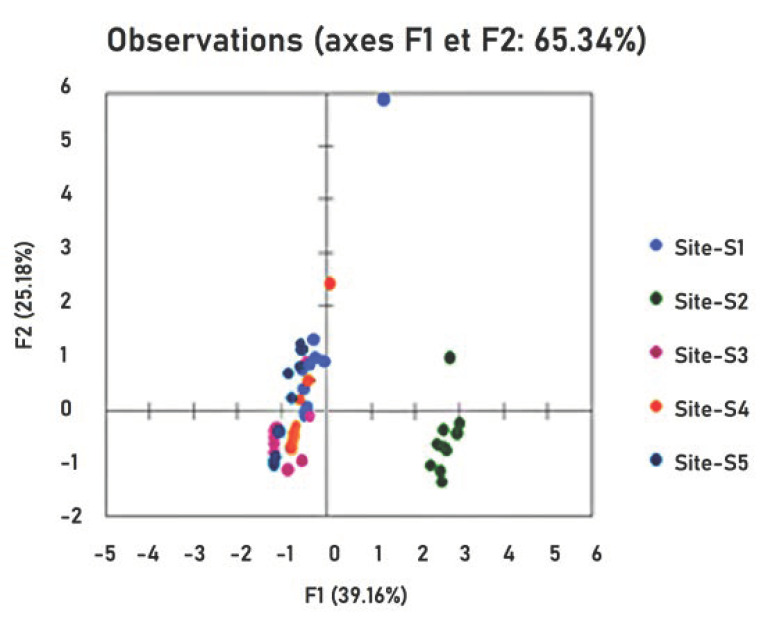
Correlation circle between samples (seawater, sediment, plant) and sites.

Samples from Mahdia coast were collected into three groups (I, II, III) according to study zone (Figure 1). Group I, composed by samples from station 1, showed a positive correlation with seawater and sediment samples. Group II was formed by samples from three stations (3, 4 and 5) indicated neither correlation among all the samples. Group III was composed by samples from station 2, which showed a positive correlation with the first component and a negative correlation with the second component (Figure 4). Results were confirmed by matrix correlation of Pearson (Table 2) between Hg concentration in samples and sites studied; highly significative positive correlations in fish, sediment, plant and seawater were found in S2 (r *S. salpa* = 0.971; r *S. aurata* = 0.954; r sediment = 0.119; r plant = 0.063; r seawater = 0.034).

### 4.2. Health Risk Assessment

In this study two approaches were used to evaluate potential human health risks related to fish consumption. Bioconcentration factors (BCF) and biota-sediment accumulation factors (BSAF) were calculated to indicate the amount of Hg accumulated in fish and plants, seawater and sediments and comparing the results with European standard (European Commission 2006). The results obtained were showed in Table 3 and they revealed important information relating to Hg accumulation in leaves of *Posidonia oceanica*, *Sparus aurata* and *Sarpa salpa*. In particular, the bioconcentration factor was higher in fish than in *P. oceanica* (BCF *P. oceanica* = 1.79 < BCF *S. salpa* = 2.77 < BCF *S. aurata* = 3.65). The same trend was observed regarding the biota-sediment accumulation factors (BSAF *P. oceanica* = 0.53 < BSAF *S. salpa* = 1.52 < BSAF *S. aurata* = 2.39). In addition, the BCF value in *S. aurata* was higher in the study by Dominik et al. (2014) than in our study [30].

According to the literature, this ratio can be influenced by different environmental factors such as the concentration of contaminant in the water, the temperature of the water that increases the bioconcentration factor, the pH of the water and the increased salinity that leads to a decrease in BCF [31].

Ostlund et al. [32] found that dissolved organic matter (DOM) limited Hg uptake from water by fish, yielding lower BCF. Higher concentrations of Hg in the leaves were measured in the contaminated areas compared to the remote areas [33].

The second approach was to compare the Hg concentration in fish with European standards and most of the results of this study are in accordance with the legislation (1 mg/kg w.w.) for Hg by the Regulation (EC) No 1881/2006 (European-Commission 2006). Except for *Sparus aurata* and *Sarpa salpa* samples from the S2 (Rejiche) that indicated values exceed the EC legislation and this could be explained by the waste water discharged from the Other National Authorities (ONAs) near the study area (European-Commission 2006).

Table 1 shows the ranges of Hg concentrations and their relative TWI% obtained in this study. The ingestion of Hg from the analyzed fish samples from site 2 could present health risks for the average consumer at 200 g portions, which is a regular serving size according to the Mediterranean diet. This situation is alarming even if one considers that half the amount of fish is eaten per week; this weekly intake concentration is slightly higher than the TWI. It should be kept in mind that regular or excessive consumption of such fish species might exceed the recommended weekly intake (TWI). Therefore, fish consumption may contribute significantly to the intake of hazardous elements such as Hg from the environment, especially in the Mediterranean Sea.

## 5. Conclusions

Mercury pollution is present in the Mediterranean Sea and consequently in sediments and in all aquatic species that represent a source of food and proteins. However, the levels of Hg found cannot represent the state of contamination of the whole ecosystem because many variables should be considered when comparing the Hg content in fish among different sites, such as: species, trophic position, food behavior, degradation of the habitats and sources of pollution.

The concentration of Hg was preferentially accumulated in fish, following by plant, sediment and seawater. The variability of Hg levels among the different sites studied may reflect variability of Hg levels found in the environment.

Our results showed that the fish species *Sparus aurata* and *Sarpa salpa* caught in the Mediterranean Sea need to be constantly monitored due to their Hg content above all in the site 2 (Rejiche) where the Hg content was higher than the European legislative limit (EFSA 2012). However, different studies should be carried out to monitor the concentrations of Hg in different and numerous fish species in the Mediterranean Sea so that the safety of the population that feeds on fish can be guaranteed. The Hg concentrations found in our work vary slightly in the two fish species analyzed and it is important to take these variations into consideration in the health risk assessment. The estimated weekly intake, calculated on the basis of the average Hg concentration of the species studied, revealed high levels of exposure associated with the consumption of *Sparus aurata* and *Salpa sarpa*. The results of this study clearly indicate that consumers who eat fish that come from this site may be exposed to high Hg content compared to the same fish species from the other sites studied. In fact, a higher fish consumption from this area of Tunisia would be of concern in terms of the risk of detrimental health effects.

The results of our work demonstrate that Hg intake, through the consumption of certain fish, is a problem that deserves special attention.

## 6. Patents

This section is not mandatory but may be added if there are patents resulting from the work reported in this manuscript.

## Figures and Tables

**Table 1:** Hg concentrations (μg/kg), standard deviation, mean values in environmental samples (seawater, sediment, *Posidonia oceanica* leaves, *Sparus aurata* muscle tissues and *Salpa sarpa* muscle tissue) and TWI% for Hg contents in *Sparus aurata* and *Sarpa salpa*.

Environmental Samples	S1	S2	S3	S4	S5
Seawater (μg/kg) (*n* = 5 × 33)	0.32 ± 0.19	0.25 ± 0.16	0.24 ± 0.15	0.21 ± 0.12	0.19 ± 0.05
Sediment (μg/kg) (*n* = 5 × 33)	7.48 ± 5.96	5.83 ± 3.57	1.88 ± 0.83	2.43 ± 1.58	3.85 ± 3.19
Plant (μg/kg) (*n* = 5 × 12)	9.27 ± 3.10	11.74 ± 2.85	16.76 ± 4.48	5.33 ± 0.05	14.34 ± 4.45
*Sparus aurata* (μg/kg) (*n* = 5 × 6) %TWI	774.78 ± 570.71 64.6%	1853.71 ± 655.61 154.5%	438.54 ± 208.76 36.5%	826.16 ± 232.041 68.8%	498 ± 470.71 41.5%
*Sarpa salpa* (μg/kg) (*n* = 5 × 6) %TWI	65.11 ± 54.03 5.4%	2518.71 ± 231.8 209.8%	403.53 ± 431.61 33.6%	14.14 ± 0.46 1.2%	35.79 ± 21.69 2.9%

**Table 2:** Matrix correlation of Person between Hg concentration in samples and sites studied.

Variables	Seawater	Sediment	Plant	*S. aurata*	*S. salpa*	Site-S1	Site-S2	Site-S3	Site-S4	Site-S5
seawater	1	0.304	0.032	0.050	0.053	0.138	0.034	−0.018	−0.067	−0.087
sediment	0.304	1	−0.065	0.192	0.082	0.424	0.119	−0.298	−0.212	−0.034
plant	0.032	−0.065	1	−0.154	0.181	−0.169	0.063	0.449 ***	−0.632	0.289
*S. aurata*	0.050	0.192	−0.154	1	0.898	−0.107	0.954 ***	−0.428	−0.049	−0.370
*S. salpa*	0.053	0.082	0.181	0.898	1	−0.273	0.971 ***	−0.109	−0.300	−0.290

*** *p* < 0.05.

**Table 3:** Bioconcentration factors (BCF) and biota-sediment accumulation factors (BSAF) in leaves of *Posidonia oceanica*, *Sparus aurata* and *Sarpa salpa*.

	*Posdidonia oceanica*				*Sparus aurata*				*Sarpa salpa*			
	BCF		BSAF		BCF		BSAF		BCF		BSAF	
Site	Mean ± SD	Min–Max	Mean ± SD	Min–Max	Mean ± SD	Min–Max	Mean ± SD	Min–Max	Mean ± SD	Min–Max	Mean ± SD	Min–Max
S1	1.65 ± 0.48	0.78–2.41	0.19 ± 0.36	<0.01–0.85	3.52 ± 0.29	3.02–4.09	2.03 ± 0.29	1.88–2.80	2.34 ± 0.55	1.36–3.28	0.87 ± 0.65	0.01–1.98
S2	1.82 ± 0.45	0.98–2.45	0.45 ± 0.36	0.06–1.12	4.02 ± 0.43	3.26–4.58	2.65 ± 0.34	2.22–3.25	4.16 ± 0.44	3.37–4.74	2.78 ± 0.35	2.38–3.41
S3	1.98 ± 0.40	1.38–2.25	0.92 ± 0.28	0.48–1.36	3.45 ± 0.44	4.58–2.68	2.39 ± 0.22	2.13–2.76	3.10 ± 0.56	2.23–4.04	2.05 ± 0.59	1.27–2.84
S4	1.52 ± 0.36	0.85–2.09	0.44 ± 0.34	<0.01–0.79	3.71 ± 0.36	3.04–4.28	2.63 ± 0.34	1.98–2.97	1.95 ± 0.36	1.29–2.52	0.8 ± 0.66	0.23–1.22
S5	1.96 ± 0.46	1.17–2.57	0.66 ± 0.43	0.18–1.56	3.53 ± 0.40	2.87–4.06	2.23 ± 0.40	1.72–3.02	2.33 ± 0.45	1.69–3.06	1.02 ± 0.50	0.34–2.03
Mean	1.79		0.53		3.65		2.39		2.77		1.52	

## Data Availability

Not applicable.

## References

[1] Alcoverro T., Cerbiān E., Ballesteros E. (2001). The photosynthetic capacity of the seagrass *Posidonia oceanica*: Influence of nitrogen and light. J. Exp. Mar. Biol. Ecol..

[2] Kim B.-M., Lee B.-E., Hong Y.-C., Park H., Ha M., Kim Y.-J., Kim Y., Chang N., Kim B.-N., Oh S.-Y. (2011). Hg levels in maternal and cord blood and attained weight through the 24 months of life. Sci. Total Environ..

[3] Zhang K., Su J., Xiong X., Wu X., Wu C., Liu J. (2016). Microplastic pollution of lakeshore sediments from remote lakes in Tibet plateau, China. Environ. Pollut..

[4] Coelho J., Pimenta J., Gomes R., Barroso C., Pereira M., Pardal M., Duarte A. (2006). Can *Nassarius reticulatus* be used as a bioindicator for Hg contamination? Results from a longitudinal study of the *Portuguese coastline*. Mar. Pollut. Bull..

[5] Beldowska M., Falkowska L. (2016). Hg in marine fish, mammals, seabirds, and human hair in the coastal zone of the southern Baltic. Water Air Soil Pollut..

[6] Maulvault A.L., Custódio A., Anacleto P., Repolho T., Pousão P., Nunes M.L., Diniz M., Rosa R., Marques A. (2016). Bioaccumulation and elimination of Hg in juvenile seabass (*Dicentrarchus labrax*) in a warmer environment. Environ. Res..

[7] Cammilleri G., Vazzana M., Arizza V., Giunta F., Vella A., Dico G.L., Giaccone V., Giofrè S.V., Giangrosso G., Cicero N. (2018). Hg in fish products: What’s the best for consumers between bluefin tuna and yellowfin tuna?. Nat. Prod. Res..

[8] Murata Y., Finkelstein D.B., Lamborg C.H., Finkelstein M.E. (2019). Tuna Consumption, Hg Exposure, and Knowledge about Hg Exposure Risk from Tuna Consumption in University Students. Environ. Toxicol. Chem..

[9] Genchi G., Sinicropi M.S., Carocci A., Lauria G., Catalano A. (2017). Hg Exposure and Heart Diseases. Int. J. Environ. Res. Public Health.

[10] Licata P., Trombetta D., Cristani M., Naccari C., Martino D., Caló M., Naccari F. (2005). Heavy metals in liver and muscle of bluefin tunA (*Thunnus thynnus*) caught in the Straits of Messina (Sicily, Italy). Environ. Monit. Assess..

[11] Parrino V., Costa G., Cannavà C., Fazio E., Bonsignore M., Concetta S., Piccione G., Fazio F. (2019). Flow cytometry and micro-Raman spectroscopy: Identification of hemocyte populations in the mussel *Mytilus galloprovincialis* (Bivalvia: Mytilidae) from Faro Lake and Tyrrhenian Sea (Sicily, Italy). Fish Shellfish Immunol..

[12] Xu X., Yekeen T.A., Xiao Q., Wang Y., Lu F., Huo X. (2013). Placental IGF-1 and IGFBP-3 expression correlate with umbilical cord blood PAH and PBDE levels from prenatal exposure to electronic waste. Environ. Pollut..

[13] Clarkson T.W., Magos L. (2006). The toxicology of Hg and its chemical compounds. Crit. Rev. Toxicol..

[14] Magos L., Clarkson T.W. (2006). Overview of the clinical toxicity of Hg. Ann. Clin. Biochem..

[15] Guzzi G., La Porta C.A. (2008). Molecular mechanisms triggered by Hg. Toxicology.

[16] Turco V.L., Di Bella G., Furci P., Cicero N., Pollicino G., Dugo G. (2013). Heavy metals content by ICP-OES in Sarda sarda, *Sardinella aurita* and *Lepidopus caudatus* from the Strait of Messina (Sicily, Italy). Nat. Prod. Res..

[17] Salvo A., Potorti A.G., Cicero N., Bruno M., Turco V.L., Di Bella G., Dugo G. (2014). Statistical characterisation of heavy metal contents in *Paracentrotus lividus* from Mediterranean Sea. Nat. Prod. Res..

[18] Giangrosso G., Cammilleri G., Macaluso A., Vella A., D’Orazio N., Graci S., Dico G.M.L., Galvano F., Giangrosso M., Ferrantelli V. (2016). Hair Hg Levels Detection in Fishermen from Sicily (Italy) by ICP-MS Method after Microwave-Assisted Digestion. Bioinorg. Chem. Appl..

[19] Conti M.E., Cecchetti G. (2003). A biomonitoring study: Trace metals in algae and molluscs from Tyrrhenian coastal areas. Environ. Res..

[20] Mergler D., Anderson H.A., Chan L.H.M., Mahaffey K.R., Murray M., Sakamoto M., Stern A.H. (2007). Methylmercury exposure and health effects in humans: A worldwide concern. Ambio.

[21] Parrino V., Kesbiç O.S., Acar Ü., Fazio F. (2020). Hot pepper (*Capsicum* sp.) oil and its effects on growth performance and blood parameters in rainbow trout (*Oncorhynchus mykiss*). Nat. Prod. Res..

[22] Mezghani-Chaari S., Hamza A., Hamza-Chaffai A. (2011). Hg contamination in human hair and some marine species from Sfax coasts of Tunisia: Levels and risk assessment. Environ. Monit. Assess..

[23] Ben Salem Z., Ayadi H. (2017). First investigation of trace metal distribution in surface seawater and copepods of the south coast of Sfax (Tunisia). Environ. Sci. Pollut. Res. Int..

[24] Bhagure G.R., Mirgane S.R. (2011). Heavy metal concentrations in groundwaters and soils of Thane Region of Maharashtra, India. Environ. Monit. Assess..

[25] Khemis I.B., Aridh N.B., Hamza N., M’Hetli M., Sadok S. (2017). Heavy metals and minerals contents in pikeperch (*Sander lucioperca*), carp (*Cyprinus carpio*) and flathead grey mullet (*Mugil cephalus*) from Sidi Salem Reservoir (Tunisia): Health risk assessment related to fish consumption. Environ. Sci. Pollut. Res. Int..

[26] Zrelli S., Amairia S., Chaabouni M., Oueslati W., Chine O., Nachi Mkaouar A., Cheikhsbouii A., Ghorbel R., Zrelli M. (2021). Contamination of Fishery Products with Hg, Cadmium, and Lead in Tunisia: Level’s Estimation and Human Health Risk Assessment. Biol. Trace Elem. Res..

[27] Maggi C., Berducci M.T., Bianchi J., Giani M., Campanella L. (2009). MethylHg determination in marine sediment and organisms by Direct Hg Analyser. Anal. Chim. Acta.

[28] Carbonell G., Bravo J.C., Fernández C., Tarazona J.V. (2009). A new method for total Hg and methyl Hg analysis in muscle of seawater fish. Bull. Environ. Contam Toxicol..

[29] Dominik J., Tagliapietra D., Bravo A.G., Sigovini M., Spangenberg J.E., Amouroux D., Zonta R. (2014). Hg in the food chain of the Lagoon of Venice, Italy. Mar. Pollut. Bull..

[30] Govers L.L., Lamers L.P., Bouma T.J., Eygensteyn J., de Brouwer J.H., Hendriks A.J., Huijbers C.M., van Katwijk M.M. (2014). Seagrasses as indicators for coastal trace metal pollution: A global meta-analysis serving as a benchmark, and a Caribbean case study. Environ. Pollut..

[31] Papp R., Aleksandar P., Nancy K., Richard T.-G. (2010). Pharmacokinetics of Cefovecin in squirrel monkey (*Saimiri sciureus*), rhesus macaques (*Macaca mulatta*), and cynomolgus macaques (*Macaca fascicularis*). J. Am. Assoc. Lab. Anim. Sci..

[32] Östlund U., Kidd L., Wengström Y., Rowa-Dewar N. (2011). Combining qualitative and quantitative research within mixed method research designs: A methodological review. Int. J. Nurs. Stud..

[33] Habib Z.A. (2017). Bisphosphonates in the treatment of osteoporosis: A review of skeletal safety concerns. Expert Rev. Endocrinol. Metab..

